# Resistance to targeted therapies in chronic lymphocytic leukemia: Current status and perspectives for clinical and diagnostic practice

**DOI:** 10.1038/s41375-025-02662-y

**Published:** 2025-06-24

**Authors:** Piers Blombery, Thomas Chatzikonstantinou, Marina Gerousi, Richard Rosenquist, Gianluca Gaidano, Sarka Pospisilova, Andrew W. Roberts, Richard W. Birkinshaw, Davide Rossi, Lydia Scarfo, John F. Seymour, Stephan Stilgenbauer, Adrian Wiestner, Jennifer A. Woyach, Jennifer R. Brown, Paolo Ghia, Kostas Stamatopoulos, on behalf of ERIC, the European Research Initiative on CLL, Piers Blombery, Thomas Chatzikonstantinou, Marina Gerousi, Richard Rosenquist, Gianluca Gaidano, Sarka Pospisilova, Andrew W. Roberts, Richard W. Birkinshaw, Davide Rossi, Lydia Scarfo, John F. Seymour, Stephan Stilgenbauer, Adrian Wiestner, Jennifer A. Woyach, Jennifer R. Brown, Paolo Ghia, Kostas Stamatopoulos

**Affiliations:** 1 Clinical Haematology (Peter MacCallum Cancer Centre/Royal Melbourne Hospital), Melbourne, VIC Australia; 2 Sir Peter MacCallum Department of Oncology, University of Melbourne, Melbourne, VIC Australia; 3 Collaborative Centre for Genomics Cancer Medicine, University of Melbourne, Melbourne, VIC Australia; 4 Institute of Applied Biosciences, Centre for Research and Technology Hellas, Thessaloniki, Greece; 5 Department of Molecular Medicine and Surgery, Karolinska Institutet, Stockholm, Sweden; 6 Clinical Genetics and Genomics, Karolinska University Hospital, Stockholm, Sweden; 7 Division of Hematology, Department of Translational Medicine, University of Eastern Piedmont and Maggiore Charity Hospital, Novara, Italy; 8 Department of Internal Medicine, Hematology and Oncology, and Institute of Medical Genetics and Genomics, University Hospital Brno and Medical Faculty, Masaryk University, Brno, Czech Republic; 9 Central European Institute of Technology, Masaryk University, Brno, Czech Republic; 10 Blood Cells and Blood Cancers Division, Walter and Eliza Hall Institute of Medical Research, Melbourne, VIC Australia; 11 Faculty of Medicine, Dentistry and Health Sciences, The University of Melbourne, Melbourne, VIC Australia; 12 Structural Biology Division, Walter and Eliza Hall Institute of Medical Research, Melbourne, VIC Australia; 13 Clinic of Hematology, Oncology Institute of Southern Switzerland, Bellinzona, Switzerland; 14 Institute of Oncology Research, Laboratory of Experimental Hematology, Bellinzona, Switzerland; 15 Faculty of Biomedical Sciences, Università della Svizzera Italiana, Lugano, Switzerland; 16 Università Vita Salute San Raffaele, Milano, Italy; 17 Strategic Research Program on CLL, IRCCS Ospedale San Raffaele, Milano, Italy; 18 Comprehensive Cancer Center Ulm (CCCU), Department of Internal Medicine III, University Hospital Ulm, Ulm, Germany; 19 NIH, National Heart Lung Blood Institute, Bethesda, MD USA; 20 Division of Hematology, Department of Internal Medicine, The Ohio State University, Columbus, OH USA; 21 Department of Medical Oncology Dana-Farber Cancer Institute, Boston, MA USA

**Keywords:** Chronic lymphocytic leukaemia, Genetics research

## Abstract

The integration of BTK and BCL2 inhibitors into the treatment of patients with chronic lymphocytic leukemia (CLL) represents a paradigm shift and has led to significant improvements in clinical outcomes, including prolonged survival and enhanced quality of life. However, despite the efficacy of these agents, resistance to targeted therapy remains a major challenge, ultimately resulting in treatment failure and disease progression for a significant proportion of patients. Related to this, diagnostic testing for genetic variants associated with resistance, such as mutations in *BTK, PLCG2* and *BCL2*, may become an increasingly common part of clinical routine practice. Addressing the need for placing the current knowledge in context, here we summarize the evidence from clinical studies and examine the underlying biology of both genetic and non-genetic resistance. Furthermore, we outline methodological approaches for the detection of gene alterations associated with targeted therapy resistance, discuss how to interpret these findings and highlight interpretation challenges. Finally, we offer insights into the clinical relevance of identifying genetic resistance to inform personalized treatment strategies and improve patient outcomes.

## Introduction

Targeted therapies using BTK inhibitors (BTKi) and/or BCL2 inhibitors (BCL2i) represent a paradigm shift in the management of patients with chronic lymphocytic leukemia (CLL) and have led to significant improvements in patient outcomes. Despite their efficacy, resistance to these targeted therapies may occur, resulting in treatment failure and disease progression for a significant proportion of patients. The specific resistance mechanisms observed and the timing of their onset show significant variation and are the result of an interplay of many factors including intrinsic disease characteristics, exposure to previous therapies and duration of exposure to the targeted agent (i.e. continuous versus time-limited therapy).

Understanding mechanisms of resistance to targeted agents may be highly informative for tailoring treatment both at the individual patient level as well as for clinical trials. Moreover, research into resistance mechanisms can impart crucial insights into disease and drug biology which may, in turn, inform the development of future therapeutic agents and approaches. In addition, diagnostic testing for genomic variants associated with resistance is becoming an increasingly common part of routine clinical practice. Herein, we summarize the landscape of resistance mechanisms observed during targeted treatment of CLL and their relevance for current clinical practice. We also provide a practical perspective on the approaches for the detection and interpretation of genomic alterations in the diagnostic laboratory that may help standardize practice in clinical trials and potentially in future clinical practice.

## Btk-directed therapies

CLL cell survival is highly dependent on tonic signaling from the B cell receptor (BcR) which is transmitted through an intracellular signaling pathway with the tyrosine kinase BTK playing a critical role. As such, disrupting BTK function in CLL (which can be achieved through targeted inhibitor therapy or targeted degradation) has emerged as a highly effective therapeutic approach.

While most patients treated with BTK inhibitors (BTKis) will achieve at least a partial response (PR) [1–4], an initial worsening of lymphocytosis concurrent with shrinking of nodal disease, captured as PR with lymphocytosis (PR-L), is common with BTKi and due to redistribution of CLL cells from lymph nodes to the blood [5]. Importantly, initial progressive and persistent lymphocytosis in this context is not an indicator of resistance [6] and usually wanes after a few months of treatment. Primary resistance to BTKi treatment is very rare in frontline-treated patients and remains relatively uncommon even in heavily pretreated patients and should prompt a reevaluation of the diagnosis (i.e. presence of Richter transformation) [7–10].

Disease progression after an initial and prolonged response to BTK inhibition is the more typically observed clinical scenario. CLL progression can happen after discontinuing BTKi treatment (e.g. due to toxicity) or in the context of continuous BTKi treatment (i.e., secondary resistance) [11]. Relapses occurring after BTKi discontinuation do not necessarily indicate secondary resistance. In fact, patients with good response to BTKi therapy who then discontinue due to adverse events can enjoy prolonged stability without progression.

### Mechanisms of resistance

#### *BTK* variants

One of the most common on-target resistance mechanisms to BTK directed therapies is the acquisition of genomic variants within the CLL cell which alter the amino acid sequence of the BTK protein and affect the binding/activity of various BTK directed therapies. The incidence of acquired *BTK* variants (which may be observed at a range of cancer cell fraction [CCF]) at progression on BTKi therapy varies between 10-80% depending on clinical context, mode of progression and the specific BTKi used (Table 1).

**Table 1:** Incidence and characteristics of *BTK* and *PLCG2* mutations in patients with CLL both within and outside clinical trials.

Reference (PMID/PMCID/DOI)	Type of study	Drug(s)	Disease setting	*BTK* mutations	*PLCG2* mutations
34019713	Observational studies	IBR	RR	80% (32/40) *only BTK*^*C481S*^ *was assessed*	Not assessed in all patients *1 in a patient without BTK mutations*
• 28418267 • (*PCYC1102, PCYC1109, PCYC1113 RESONATE*)	Clinical trial^a^	IBR	RR	78.3% (36/46)	15.2% (7/46) *4 in patients without BTK mutations*
38754046 (*ELEVATE-RR*)	Clinical trial^b^	IBR, ACA	RR	ACA: 66% (31/47) IBR: 37% (11/30)	ACA: 6% (3/47)^c^ IBR: 20% (6/30)^c^
• 30508305	Observational studies	IBR, ACA	NA	65.6% (19/29)	0% (0/29)
• 36696464	Observational studies	IBR	TN & RR	61.2% (30/49) *6 detected only with ddPCR*	28.5% (14/49) *2 in patients without BTK mutations*
• 32726539	Clinical trial^d^	IBR	TN	50% (6/12)	58.3% (7/12) *4 in patients without BTK mutations*
• 38313250	Observational studies	IBR, ACA PIR, VEC	TN & RR	−IBR, & ACA: 41.7% (15/36) PIR & VEC: 83.3% (5/6)	−IBR, & ACA: 8.3% (3/36)^e^ PIR & VEC: 33.3% (2/6)
• 37314786 • (*PCYC1122e, RESONATE, RESONATE-2, RESONATE-17 iLLUMINATE*)	Clinical trial^f^	IBR	TN & RR	−25% (3/12) in TN pts 49% (22/45) in RR pts	−8% (1/12) in TN pts 13% (6/45) in RR pts
10.1182/blood-2023-173547 (*ALPINE*)	Clinical trial^g^	IBR, ZANU	RR	ZANU: 20.8% (5/24) IBR: 10.7% (3/28)	ZANU: 0% (0/24) IBR: 7.1% (2/28)^h^
PMC10428413 (*BRUIN*)	Clinical trial^i^	PIR	RR	55.5% (27/49)	8% (4/49)

*IBR* ibrutinib, *ACA* acalabrutinib, *PIR* pirtobrutinib, *VEC* vecabrutinib, *RR* relapsed/refractory, *TN* treatInaive, *NA* not available, *ddPCR* Droplet Digital polymerase chain reaction.

^a^NCT01105247 (phase 2), NCT01217749 (phase 2), NCT01589302 (phase 2), NCT01578707 (phase 3); ^b^NCT02477696 (ELEVATE-RR, phase 3); ^c^2 in patients without BTK mutations in each arms.

^d^NCT01500733 (phase 2 trial); ^e^All co-occurred with BTK mutations; ^f^NCT01500733 (phase 2), NCT01722487 (RESONATE-2, phase 3), NCT02264574 (iLLUMINATE, phase 3), NCT01744691 (phase 2), NCT01578707 (RESONATE, phase 3); ^g^NCT03734016 (ALPINE, phase 3); ^h^One patient without BTK mutations; ^i^NCT03740529 (BRU½ phase 1/2).

**Fig. 1:**
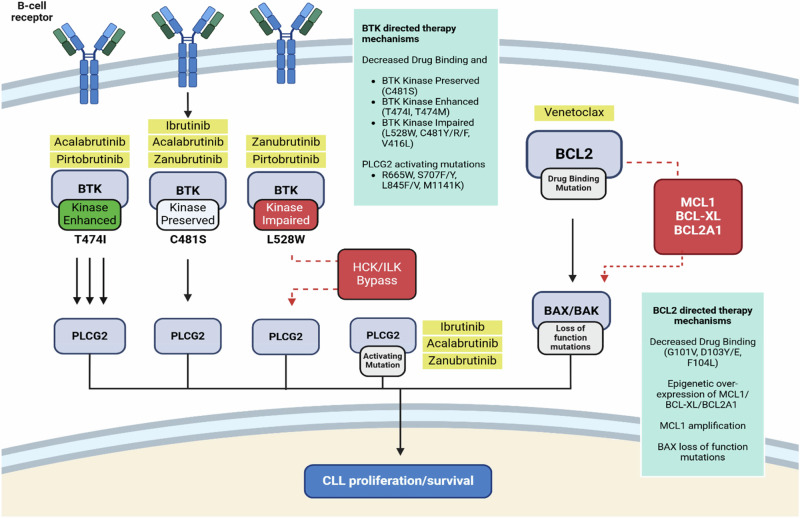
Overview of genomic resistance mechanisms to targeted therapy in CLL. Targeted agents are highlighted in yellow and target proteins in gray. The light green boxes provide a summary of genomic resistance mechanisms to targeted agents.

Acquired variants in *BTK* observed in the context of BTK-directed therapies can broadly be considered in three main groups (Fig. 1):

(i) Variants that alter drug binding but have little to no effect on the kinase function of the BTK protein. This group includes *BTK* Cys481Ser, the first and most common BTK inhibitor resistance variant described, which converts the covalent interaction between ibrutinib (and indeed other covalent BTKis) and BTK to a reversible non-covalent interaction. This allows ATP to re-compete with covalent BTK inhibitors due to their short plasma half-life, which re-establishes enzyme activity and downstream signaling [12, 13].
(ii) Variants that alter drug binding but also disrupt normal BTK kinase function. Examples of this group of variants (variably termed ‘kinase-impaired’ or ‘kinase-dead’) include *BTK* Leu528Trp, Cys481Arg and Val416Leu [14, 15]. Whilst the precise mechanism through which BTK signaling is preserved in the presence of these kinase-impaired *BTK* variants is still being elucidated, experimental data to date strongly support that these variants induce a scaffolding neofunction involving BTK that results in novel interactions with other intracellular signaling kinases (HCK and ILK) to re-establish downstream signaling [14, 16]. Importantly, the phenomenon of ‘kinase-impaired’ variants being associated with bypassing mechanisms involving alternative intracellular kinases has been observed in other contexts, most notably kinase-impaired BRAF variants [17].
(iii) The final group of variants are those affecting the Thr474 codon (most commonly Thr474Ile). Thr474 functions as a gatekeeper residue controlling access to the catalytic domain and mutations at this position disrupt a hydrogen network between multiple amino acids resulting in decreased ability of covalent and non-covalent inhibitors to bind to BTK [18]. Indeed, the *BTK* Thr474Ile is paralogous to *ABL1* Thr315Ile [19], the gatekeeper mutation conferring TKI resistance in CML. In addition, Thr474 mutations have been observed to increase BTK kinase activity in in vitro models [14, 20] however the precise biological and clinical implications of this increased kinase activity is unclear. Notably an increased intrinsic kinase activity is also observed with *ABL1* Thr315Ile [21, 22]. Co-occurrence of Thr474Ile and Cys481 mutations have been observed in clinical cohorts, primarily thus far in patients treated with acalabrutinib [23].

**Fig. 2:**
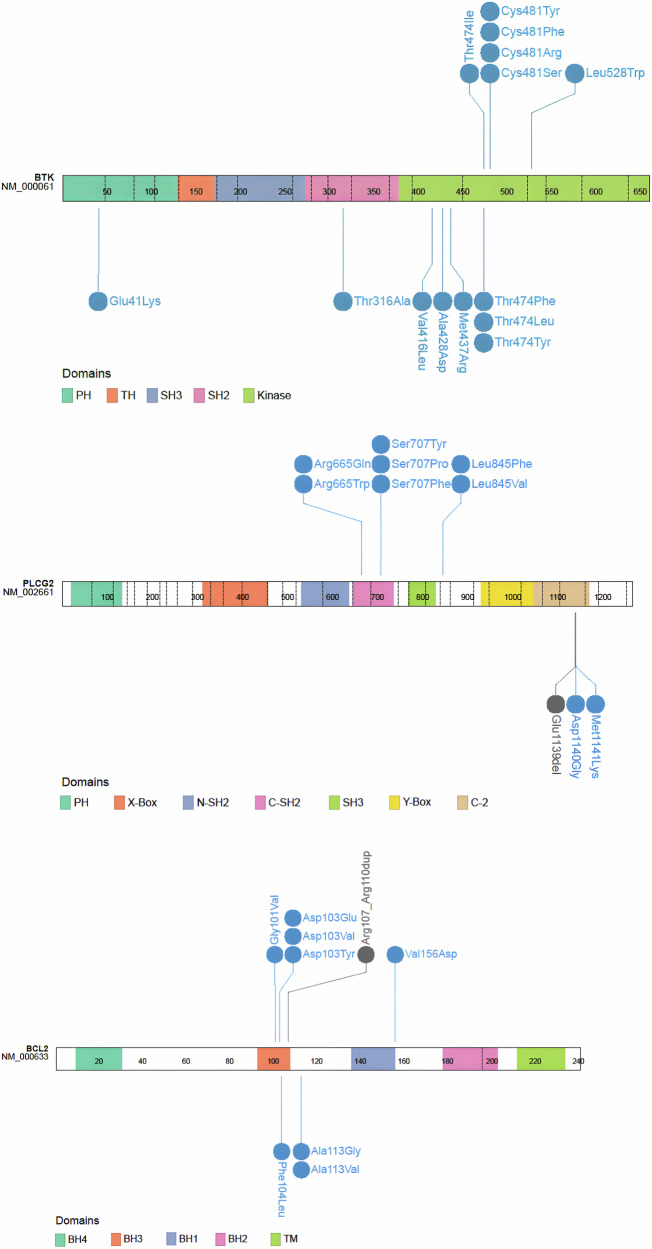
Common resistance variants observed in the context of targeted therapy with BTK inhibitors or BCL2 inhibitors. Figure 2a depicts variants observed in *BTK*, Fig. 2b in *PLCG2*, and Fig. 2c in *BCL2*. Variants are indicated by blue (missense) or gray (in-frame changes) lollipops positioned on a linear schematic of the protein sequence with domains annotated. Figure created by ProteinPaint (Zhou et al., Nat Genet 2016; PMID: 26711108).

Commonly observed acquired *BTK* variants are summarized in Table 2 and Fig. 2.

**Table 2:** Common sequence variants and functional characteristics associated with targeted therapy resistance in CLL.

Gene	Variant	Comment
*BTK*	Cys481Ser	Kinase-preserved; Decreased binding affinity of C481S for cBTKi
	Cys481Arg/Phe/Tyr	Kinase-impaired [3, 4]; Decreased inhibition of BTK auto-phosphorylation by cBTKi [4]
	Leu528Trp	Kinase-impaired [3, 28]; Decreased binding affinity of L528W for both cBTKi and ncBTKi [3]; clinical resistance to ncBTKi and zanubrutinib
	Thr474Ile/Met	Kinase-enhanced [3, 9]; In vitro resistance to cBTKi [74] clinical resistance to acalabrutinib and ncBTKi
	Val416Leu	Kinase-impaired [3]; Observed in ncBTKi progression [28]; In vitro resistance to ncBTKi [75]
	Ala428Asp	Kinase-impaired [3]; Observed in ncBTKi and BTK degrader progression [28, 33]
*PLCG2*	Arg665Trp	Hyper-responsiveness to upstream signaling [13]
	Ser707Tyr	Hyper-responsiveness to upstream signaling [76, 77]; Causative variant for APLAID syndrome in germline context [78]
	Leu845Phe	Hyper-responsiveness to upstream signaling [13]
	Asp993His	Hyper-responsiveness to upstream signaling [79, 80]; Causative variant for APLAID syndrome in germline context [79]
	Met1141Lys	Hyper-responsiveness to upstream signaling [81]; Causative variant for APLAID syndrome in germline context [81]
BCL2	Gly101Val	Decreases affinity of venetoclax for BCL2 [40]
	Asp103Tyr	Decreases affinity of venetoclax for BCL2 [42, 43]
	Asp103Glu	Decreases affinity of venetoclax for BCL2 [41]; Retained affinity and sensitivity for navitoclax [41]
	Phe104Leu/Ile	Decreases affinity of venetoclax for BCL2 [40, 44, 82]
	Val156Asp	Decreases affinity of venetoclax for BCL2 [42]

*cBTKi* covalent BTK inhibitor, *ncBTKi* non-covalent BTK inhibito, *APLAID* autoinflammation-PLCG2-associated antibody deficiency.

#### Gain of function PLCG2 variants

Numerous missense variants have been observed in *PLCG2* in the context of BTKi therapy including Arg665, Ser707, Leu845 and Met1141 codons (Table 2 and Fig. 2). PLCG2 is the direct downstream target of BTK and these variants result in hypermorphic PLCG2 function by both potentially constitutive activation as well as hyper-sensitivity to upstream signaling (Fig. 1) [24]. Indeed, many of the somatic *PLCG2* variants observed in the context of BTKi therapy overlap with those occurring in autoimmune PLCG2-associated immune dysregulation (APLAID), an inherited syndrome caused by germline gain-of-function *PLCG2* variants [25]. Notably, *PLCG2* variants are rarely seen alone but rather more frequently observed in conjunction with *BTK* variants, commonly at very low CCF [12]. Whether and how often these variants occur in the same cell as BTK variants has yet to be definitively established.

#### Genomic and non-genomic alterations beyond the BcR pathway

A variable proportion of patients with secondary resistance to BTK directed therapies have no detectable *BTK*/*PLCG2* resistance variants (Table 1). Multiple potential mechanisms have been proposed to be mediating resistance in the *BTK/PLCG2* wildtype setting, including variants in *EGR2* and NF-κB pathway genes along with deletions of chromosomes 2p and 8p [26–28]. Activated AKT together with deregulated PTEN and FOXO3a have been previously observed in ibrutinib-resistant CLL cells [29] as well as GAB1 upregulation leading to tonic AKT activity and increased homing capacity [30]. In addition, a “functional resistance” is thought to result from decreased dependence on proximal BcR signaling or its bypass through other pathways including the mitogen-activated protein kinase (MAPK) pathway [31] and the Toll-like receptor (TLR) pathways [31–33]. The precise frequency and contribution of these abnormalities to disease resistance and their specificity for BTK-directed therapies require further study.

#### Tumor microenvironment

Ibrutinib treatment has also been shown to have a negative impact on the anti-tumor properties of nurse-like cells (NLCs) in the tumor microenvironment (TME) which displayed reduced phagocytic ability, while expressing immunosuppressive cytokines, overall preventing ibrutinib-mediated primary CLL cell apoptosis [34, 35]. Furthermore, treatment of CLL cells with ibrutinib and venetoclax, after coculturing the tumor cells with TME agonists such as interleukin-10 (IL-10), CD40L, and CpG-ODNs (TLR-9 specific agonists), led to the activation of the NF-κB signaling pathway (especially alternative NF-κB). Subsequently, it induced the expression of the anti-apoptotic proteins MCL-1 and BCL-XL, promoting resistance to the combination therapy [36].

### Covalent BTK inhibitors

The covalent BTKis (acalabrutinib, ibrutinib and zanubrutinib) share a similar mechanism of action in that they all bind irreversibly (covalently) to the Cys481 residue of BTK. Ibrutinib was the first agent in this class to enter clinical use which was followed by acalabrutinib and zanubrutinib with increasing kinome selectivity for BTK over other related kinases [37].

The pattern of *BTK* resistance variants observed differs significantly across the different covalent BTKis. *BTK* Cys481Ser is the dominant resistance variant observed in patients treated with ibrutinib, while kinase-impaired and Thr474 variants are more rarely observed [23]. In contrast, whilst Cys481Ser is the most frequently observed variant in patients with disease resistant to acalabrutinib, these patients also have a significantly higher incidence of Thr474 variants (typically observed in conjunction with a Cys481Ser variant) [23]. Finally, the *BTK* variant profile of zanubrutinib and tirabrutinib resistance differs again with Cys481Ser still being most common, but significantly higher rates of kinase-impaired *BTK* variants (commonly *BTK* Leu528Trp) than either ibrutinib or acalabrutinib [38, 39].

### Non covalent BTK inhibitors

The non-covalent BTK inhibitors (ncBTKi) were designed to overcome the loss of covalent BTKi binding site that results from BTK Cys481 variants [40]. Pirtobrutinib is currently the most advanced ncBTKi clinically, having received accelerated approval from the FDA in December 2023 for patients with CLL after prior BTKi and venetoclax exposure. The BRUIN trial confirmed a high response rate (82%) in relapsed/refractory CLL including in those with Cys481Ser variants (comprising 38% of the cohort) [10]. Patients with disease progressing on pirtobrutinib in the BRUIN study showed a relatively high rate of acquired kinase-impaired BTK variants, particularly Thr474 and Leu528Trp [27/49 (55.5%) of tested cases with clinical progression] [41, 42]. Other ncBTKi have been studied clinically including vecabrutinib and nemtabrutinib, however, there have been no reports of *BTK* variants at clinical progression on these agents to date.

### BTK “degrader” therapy

An emerging class of BTK-directed therapies are the proteolysis-targeting chimeras (PROTACs) which result in selective degradation of the BTK protein through complexing with E3 ligases [43]. In this way, these agents offer an attractive mechanism for overcoming various *BTK* resistance variants observed on cBTKi or ncBTKi therapies [14]. Several agents are under evaluation (NX-5948, NX-2127, BGB-16673) and have shown encouraging safety and efficacy in phase 1 trials among heavily pretreated patients with CLL after BTKi and BCL2i exposure [44, 45]. Interestingly, one patient treated with BGB-16673 developed a *BTK* Ala428Asp variant [46], however further data are required to understand the landscape of *BTK* variants occurring on these agents.

## Resistance To Bcl2 Inhibitors

CLL cells show very high expression of the pro-survival molecule BCL2 and are critically reliant on this mechanism to avoid apoptotic cell death [47]. Small molecules that bind specifically to BCL2, relieving restraints on apoptosis in CLL cells, represent a major advancement in the treatment of CLL and have dramatically improved patient outcomes in the relapsed refractory [48, 49] and treatment naïve settings [50]. The most commonly used agent in this class is venetoclax and whilst newer agents are currently being clinically evaluated (including sonrotoclax [51] and lisaftoclax [52]), to date the majority of our understanding of BCL2i resistance comes from patients treated with venetoclax.

Similar to BTKi, true primary resistance to venetoclax-containing regimens is very rare in the first line setting and is even rarer in relapsed/refractory disease, and should raise consideration of the potential for Richter transformation. Indeed, the great majority of patients achieve significant cytoreduction unless treatment is ceased early due to toxicity [48, 50, 53–56]. Only in early monotherapy trials for previously heavily treated patients has failure to achieve at least a partial response been seen in more than 10% of patients [48, 56, 57]. Consequently, secondary resistance is the predominant form of resistance observed clinically.

Studies on resistance mechanisms to venetoclax are primarily based on the analysis of samples from patients treated with continuous venetoclax [58–60]. Presently, very little is known about clonal evolution during time-limited venetoclax therapy which achieves deep and durable remissions. Limited data on the success of retreating with venetoclax after initial time-limited therapy would also suggest that relapsing disease is not a priori resistant to venetoclax.

Studies based on samples from patients progressing on continuous venetoclax therapy have demonstrated that emergent resistance to BCL2 inhibition can arise through distinct genetic and epigenetic changes [58, 61–64]. The recurring mechanisms observed commonly across patients are outlined below. Similar to the BTKi context, a recurrent finding is that clinical resistance to venetoclax within an individual patient is often multifactorial with different mechanisms operating in CLL subpopulations collected from the same patient [59, 60, 62, 65]. Indeed, single cell multi-omics studies indicate that within single cells often two mechanisms may occur concurrently [60].

### Mechanisms of resistance

#### BCL2 variants

**Fig. 3:**
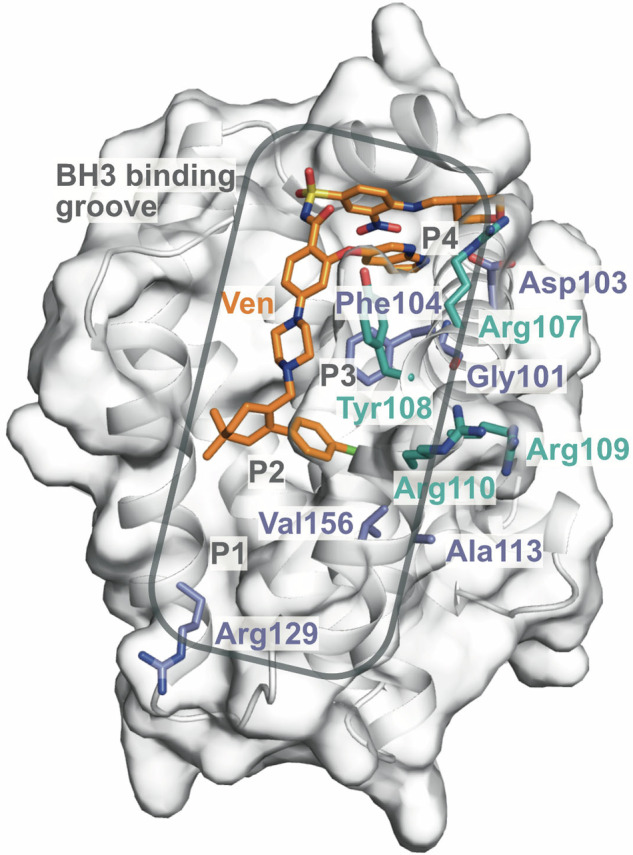
Surface representation of the BCL2 bound to venetoclax crystal structure (PDB 6O0K) showing residues that are mutated in venetoclax therapy resistance. Substitution residues are in blue, duplicated residues in teal, and venetoclax in orange interacting with the BCL2 hydrophobic pockets (P2 and P4), which are indicated in grey. The BH3 binding groove, consisting of hydrophobic pockets (P1-4), is indicated by the gray box.

Similar to variants arising in *BTK* on BTKi therapy, variants in *BCL2* that affect drug binding are the canonical direct resistance mechanism observed in patients with CLL treated with continuous venetoclax (Table 2 and Fig. 2). *BCL2* variants that have been observed to emerge in patients with CLL progressing on venetoclax in several independent clinical cohorts include Gly101Val [61], Asp103Tyr/Glu [62, 66, 67] and Phe104Leu/Ile/Cys [68], with experimental evidence supporting decreased drug binding but retention of pro-survival function. Analysis of crystal structures of the Gly101Val variant suggests that these mediate decreased venetoclax-BCL2 binding through a knock-on effect of Val101 to a three-dimensionally adjacent residue, Glu152 [68]. Other variants with less experimental/functional supporting data, but nevertheless occurring relatively specifically in the context of relapsed CLL treated with venetoclax, are Asp103Val, Arg107_Arg110dup, Ala113Gly, Arg129His and Val156Asp [62, 69–71]. Multiple variants can occur simultaneously within the same CLL patient. Notably, many of these variants occur either in, or adjacent to, the P4 pocket (critical for mediating venetoclax binding and selectivity over other BCL2 family molecules) (Fig. 3), or the BH3 binding groove more generally and are predicted to impair venetoclax-BCL2 binding [68].

#### Altered expression of proteins involved in apoptosis

Outside of drug-binding *BCL2* variants, other direct resistance mechanisms to BCL2 inhibitor therapy involve perturbations of other proteins in the intrinsic apoptotic pathway. The most commonly observed abnormalities are increased expression of the alternative pro-survival BCL2 family molecules, MCL1, BCL-xL and BCL2A1 [58, 60, 61]. Higher expression of these proteins may occur by copy-number gain/amplification e.g. *MCL1* [58, 60, 72] but most have epigenetic origins [60]. Recent work has also uncovered further complexity of cooperating genetic lesions in the apoptosis pathway, including loss of function *PMAIP1* (NOXA) variants and loss-of-function *BAX* variants [60, 65, 69].

#### TP53 dysregulation

Loss or reduction in p53 function through TP53 aberrations (i.e. del(17p) and/or *TP53* mutation) are associated with shorter duration of response with all CIT regimens and also with targeted therapies including venetoclax monotherapy [48, 73] and venetoclax-containing combination regimens. [50, 74, 75]. Intact p53 function is not required for achievement of complete remission [48, 56] and, indeed, complete remission rates are similar between *TP53*-wildtype and *TP53*-aberrant CLL [48, 75, 76]. However, aberrant p53 function in CLL is associated with inferior prognosis compared to intact p53 with venetoclax containing regimens [50, 74]. While venetoclax acts downstream of p53 in apoptosis initiation, when the mitochondrial permeabilization triggered by BH3 mimetics is sub-lethal, mitochondrial DNA release induces p53 activation, which in turn can generate a second wave of apoptotic stimulus through induction of PUMA and NOXA, which maximizes cell killing [77]. Although p53 function does not influence the susceptibility of mitochondria to apoptosis, absence of p53 function does reduce maximal BAX/BAK activation [78]. Consequently, in the absence of p53 function, there is a higher chance of escape, especially when venetoclax concentrations are submaximal [78]. Although follow-up is shorter, it appears that the time-limited combination of venetoclax and BTK inhibition, with or without an anti-CD20 does not obviate the long-term negative prognostic impact of *TP53* aberrations in patients with treatment-I CLL [79].

### BAX clonal hematopoiesis

The CLL compartment in patients treated with BCL2i adapts through the acquisition of *BCL2* variants and alternative pro-survival molecule expression as described above. However, it has been recently observed that patients treated with BCL2i therapy may have adaptive clonal hematopoiesis outside the primary tumor compartment [80]. Hypothesized to be the result of the selective pressure of BCL2i therapy throughout the hematopoietic compartment, this is primarily manifest as loss of function *BAX* variants which may be detected in remission in patients with CLL on BCL2i with single-cell sequencing showing presence within the myeloid and NK-cell compartments [81]. Moreover, a recent analysis of patients randomized between venetoclax-obinutuzumab and chlorambucil-obinutuzumab in the CLL14 trial has shown a significant enrichment of BAX clonal hematopoiesis in the venetoclax containing arm [82]. Interestingly, *BAX* variants have also recently been recognized as a rare driver in age-related clonal hematopoiesis [83]. Whilst the potential clinical significance of BAX-mutated clonal hematopoiesis is unknown currently, it is an important phenomenon to be aware of given it may be detected during genomic testing of patients with CLL.

## Diagnostic considerations for the investigation of resistance mutations in patients treated with targeted therapy

A summary of recommendations and considerations in a clinical setting, including clinical trials and observational studies is shown in Table 3.

**Table 3:** Summary of recommendations for resistance testing in chronic lymphocytic leukemia.

Domain	Recommendation
**Testing methodology**	Targeted next generation sequencing (NGS) is the preferred methodology for assessing resistance mutations in clinical practice
	The sensitivity of the molecular methodology used should be a limit of detection of at least 5% VAF.
	Ideally all coding exons of *BTK*, *PLCG2* and *BCL2* should be targeted in NGS assays however alternatively only *BTK* exons 14, 15 and 16 (NM_000061); *PLCG2* exons 19, 20, 24 and 30 (NM_002661) and *BCL2* exon 2 (NM_000633) can be covered.
	Testing used for clinical decision making and in routine practice should be performed in an appropriate clinically accredited laboratory
**Sample considerations**	Testing of peripheral blood, bone marrow aspirate or involved lymph node can be performed if tumor burden is sufficient (ideally > 40-50% disease burden).
	Testing of samples post CD19+ enrichment may be useful in cases of low disease burden
**Reporting of variants**	Methodology, covered regions and sensitivity/limit of detection should be stated on the diagnostic report
	Disease burden of sample (as assessed by morphology or flow cytometry) should be stated on the report along with variant allele frequency of detected variants
	Common resistance variants for cBTKi include BTK Cys481Ser/Arg/Phe/Tyr, Leu528Trp, Thr474Ile and PLCG2 Ser707Tyr, Leu845Phe and Asp993His; for ncBTKi include Leu528Trp and Thr474Ile; for venetoclax include Gly101Val, Asp103Tyr/Glu, Phe104Leu and Val156Asp
	Variants should be classified according to accepted curation frameworks* and an interpretative comment that takes into account all the variants detected in the sample should be provided
**Clinical decision making**^**#**^	Testing for resistance variants is not recommended prior to first exposure to BCL2i or BTKi as primary resistance is rare
	Testing for resistance variants should be performed when the results of testing will influence patient management. Testing should be considered prior to the new line of therapy, in the context of (i) ncBTKi after previous exposure to cBTKi [41, 42] or (ii) re-treatment with BCL2i/BTKi [54, 92]
	Variants detected should be interpreted in the context of clinical progression, considering clinical features (including previous treatments) and cancer cell fraction
	Therapy providing ongoing clinical benefit should not be ceased upon detection of a resistance variant if the patient has not had definitive clinical progression
**Clinical trial considerations**	Identification and investigation of resistance mechanisms should be prioritized as part of clinical trial protocols
	Tumor samples (either PB/BM) should be collected before initiation of therapy, sequentially during the course of treatment and upon disease progression
	DNA/RNA from PB/BM at each timepoint should be stored. Consideration of live cell storage at critical timepoints (e.g. before therapy and upon progression)

*NGS* next generation sequencing, *VAF* variant allele frequency, *PB* peripheral blood, *BM* bone marrow aspirate.

*Standards and Guidelines for the Interpretation and Reporting of Sequence Variants in Cancer from the Association for Molecular Pathology, American Society of Clinical Oncology, and College of American Pathologists.

^#^The “clinical decision making” recommendations are based on the authors’ opinion. Currently, high-quality data to guide clinical decisions on how to use resistance mutations for treatment decisions are lacking.

### Methodological approaches

Multiple types of genomic alterations (sequence variants, copy-number variants (CNVs) and structural variants) may be associated with targeted therapy resistance. That said, the most frequent tested abnormalities are single nucleotide variants (SNVs) and small insertions/deletions (indels) in *BTK*, *PLCG2* and *BCL2*. Given the large number of potential nucleotide changes and often low variant allele frequency (VAF) that may give rise to relevant resistance variants in these genes, next-generation sequencing (NGS) has rapidly become the most commonly used methodology for detecting these alterations.

Targeted NGS gene panels employed by diagnostic laboratories in hematological malignancy typically have sequencing depths that permit reliable detection of variants down to approximately 1–5% VAF. Allele specific methodologies (e.g. droplet digital PCR) are potentially useful for single and high-sensitivity variant detection (potentially <0.1% VAF detection limit). However, given their absolute specificity for a single mutation they cannot be relied upon as an approach for screening patients for all potential resistance variants.

In addition, given the frequent presence of multiple resistance variants spanning increasing parts of relevant genes, NGS targeting of the whole coding region (including splice sites) is the most clinically relevant approach currently. However, if panel size is a limiting factor then *BTK* exons 14, 15 and 16 (NM_000061); *PLCG2* exons 19, 20, 24 and 30 (NM_002661) and *BCL2* exon 2 (NM_000633) will cover the majority of variants described to date. It should be noted that *BCL2* exon 2 has a high GC content and may pose challenges with primer design and suboptimal coverage in amplicon-based panels but tends to be less of an issue using capture-based panels.

NGS testing for variants in *BTK, PLCG2* and *BCL2* is ideally performed after target enrichment and usually as part of a broader panel of genes assessed. There are numerous enrichment technologies that may be used, including two-primer amplicon, single primer extension/amplicon and hybridization-based approaches. These enrichment strategies are frequently combined with unique molecular indexing and duplex identification to improve sensitivity and specificity.

Ultimately any of these technical approaches may be used, however the laboratory performing the assay should have a robust understanding of the analytical performance of their assay including, but not limited to, sensitivity, specificity, reproducibility, linearity and coefficient of variation.

### Sample considerations

When assessing for resistance variants, the most appropriate sample to test depends on both the clinical context and pattern of disease involvement. In the non-transformed setting, the most commonly tested sample is DNA extracted from mononuclear cells from peripheral blood (PB) or bone marrow (BM) aspirate due to ease of availability. DNA extracted from lymph node biopsy (either fresh or formalin-fixed, paraffin-embedded) may be used in the context of an SLL phenotype when there is no involvement of the PB or BM, or where there is discordant behavior of a specific anatomical site warranting directed evaluation.

It should be noted that in one series approximately half of patients with concurrent sampling of PB and lymph node showed differences in clonal composition between the two compartments [84]. A case report and preliminary data in a larger cohort support the possibility of similar separation of clonal evolutions between compartments in patients treated with BTKi [85]. In the same vein, *BTK* and/or *PLCG2* mutations are more often identified in patients with disease progressing with prominent leukemic disease than in patients progressing primarily with nodal disease [86].

Whilst there is no absolute threshold of disease burden that is required to perform testing, the ability to detect low-frequent variants will be compromised at lower disease burden within the sample tested. As an example, if the disease burden in the sample is 20% and the molecular method has an intrinsic sensitivity of 2% VAF, then variants will only be detected when they involve greater than 10-20% of the clonal compartment (depending on zygosity). Enrichment of tumor cells with either CD19^+^ selection or negative depletion (e.g. MACS) may be of value, and is recommended for samples with low tumor burden if detection of subclonal variants is deemed of clinical relevance for the patient. In the context of possible transformed disease, the molecular profile of the transformed compartment may be significantly different from the untransformed CLL compartment and therefore samples containing transformed disease should be tested if deemed clinically relevant.

Finally, cell-free DNA (cfDNA) extracted from patient plasma can be used as a source for testing [87, 88]. However, the current understanding of the clinical relevance of findings from this compartment is limited. In addition, the quantity of circulating tumor DNA that can be routinely extracted from plasma in patients with CLL is low and therefore lower input/more sensitive methodologies may be required to detect variants. Whilst it is theoretically possible, there is no current evidence that the spectrum of resistance variants present in cfDNA differs significantly from an appropriate sample of the tumor compartment.

### Reporting of variants

For accurate clinical reporting of laboratory test results, it is crucial to provide essential details about the sample type and the methods used for DNA extraction, target amplification and sequencing. Specifically, when reporting results from NGS-based tests, comprehensive information on the sequencing technology should be included. This should specify the type of targeted NGS applied (amplicon-based or capture-based), gene/exon coverage, the sequencing instrument used, and the laboratories validated limit of detection (LOD).

Each variant detected during diagnostic workup should be described at both the gene and protein levels adhering to HGVS nomenclature. Variant allele frequency (VAF) should be reported for assays that have been validated to report quantitative measurements. The observed VAF of any given variant(s) should be interpreted in the context of the CCF of the sample. This can be estimated by multiplying the observed VAF of an individual variant (or sum of VAFs in the case of multiple variants) by two for *BCL2, PLCG2* and *BTK* (for females) and dividing by the observed quantity of disease present in the sample (typically most accurately quantitated by flow cytometry). This correction will give an approximation of the resistant proportion of the disease compartment that is accounted for by these variants. As mentioned, a range of clonality may be observed for variants in *BTK/PLCG2/BCL2* with some patients having very low levels of identifiable resistance variants, whereas others having an almost complete clonal dominance by one particular variant. These scenarios potentially have different implications for therapeutic decision making.

After assessment using population databases (e.g. gnomAD v4) to determine germline versus somatic origin of the variant (in the context of tumor-only sequencing), each detected variant should be curated taking into account the precise molecular and predicted protein consequence (including assessment for splicing effect). Relevant literature for each variant detected should include previous peer-reviewed descriptions of emergence in the context of targeted therapy; evidence from clinical studies reporting the particular variant; the specific agent to which it is proposed as a resistance variant; and evidence from germline literature where appropriate (e.g. *BTK* loss of function variants or *PLCG2* gain of function variants). Generally, the evidence for a variant being a predictive biomarker of targeted therapy resistance should derive from randomized control trials to assess a clinical endpoint in patients with and without the variant biomarker. While this type of evidence may exist for more non-specific markers of therapy resistance (e.g. variants in *TP53* for patients treated with chemoimmunotherapy), it does not generally exist for specific on-target resistance variants arising during targeted therapy (e.g. *BTK*, *PLCG2* and *BCL2*). This should be acknowledged if diagnostic laboratories are using variant curation frameworks such as the *Standards and Guidelines for the Interpretation and Reporting of Sequence Variants in Cancer* from the Association for Molecular Pathology, American Society of Clinical Oncology, and College of American Pathologists [89].

## Clinical Implications of on-target resistance mutations

### Clinical utility of resistance variant detection in current clinical practice

How to use resistance variant testing in current clinical practice remains incompletely defined and international guidelines on this topic are currently lacking. Despite this, in a rapidly changing therapeutic landscape, with new agents emerging within classes as well as entirely new classes entering the clinic, there is likely to be a need for rapid uptake and integration of new data obtained in clinical trials as well as in real-world settings.

Primary resistance to cBTKi and BCL2 inhibitors is extremely rare clinically and resistance variants in *BTK* and *BCL2* have not been observed in CLL specimens prior to the relevant drug exposure [90]. Therefore, there is minimal value testing for *BTK*/*PLCG2*/*BCL2* variants prior to commencement of therapy. Testing performed at time of suspected or confirmed clinical progression on or after targeted therapy is more commonly performed and the results of these tests can help interpret and confirm other clinicopathological observations. That notwithstanding, therapy providing ongoing clinical benefit should not be ceased if the patient has not had definitive clinical progression, since some patients may continue to derive benefit for prolonged periods after the first detection of a resistance variant [91]. However, the detection of such variants is predictive of a higher rate of subsequent disease progression and close monitoring is appropriate. The recognition of such variants can provide a window of opportunity to plan and prepare for the next line of therapy or consider available clinical trials.

An emerging area of clinical concern is potential cross resistance between cBTKi and ncBTKi. Both acalabrutinib and zanubrutinib have a significant incidence of resistance variants (Thr474Ile and Leu528Trp respectively) that have been identified as resistance variants also to pirtobrutinib. The presence of a high CCF cross-resistant BTK variant (particularly Thr474Ile or Leu528Trp) after cBTK therapy should prompt consideration of alternative therapies to pirtobrutinib, noting that venetoclax is active in this context [54, 92]. Going forward it will be important to determine how distinct mutations associated with progression on cBTKi impact outcomes of re-targeting BTK with either ncBTKis or BTK degraders.

The efficacy of re-treatment after time-limited venetoclax (in the front line or relapsed/refractory setting) is an area of active study. Whilst the incidence of *BCL2* resistance variants is likely lower in the time-limited context [70], the presence of a high CCF/VAF *BCL2* resistance variant could potentially be integrated into decision making regarding whether to pursue re-treatment or an alternative available therapy. Appropriately designed studies will be required to definitively provide answers to these questions; however, these data are unlikely to be imminently available for integration into care of patients in the clinic today.

The detection of variants with very low VAF/CCF in patients with clinical progression indicates that other resistance mechanisms may also be in play. The clinical significance of these variants, especially when considering re-treatment with the same drug class or cross-resistance between cBTKi and ncBTKi, is currently unknown.

### Maximizing understanding of resistance from clinical studies

Identification of resistance mechanisms should be an important component of clinical trials of targeted therapies. In that regard, the comprehensive collection of clinical and laboratory characteristics within clinical trials provides an ideal setting to identify markers associated with the development of resistance and to differentiate between distinct resistance mechanisms. Ultimately, investigating resistance mechanisms in interventional clinical trials and real-world studies requires the systematic collection of appropriate samples with the following considerations:

- *Timepoints* – samples should be collected (i) at baseline (before initiation of therapy) (ii) sequentially during the course of treatment and (iii) upon disease progression.
- *Sample type* – DNA and RNA should be stored from peripheral blood (PB)/bone marrow aspirate (BM) at baseline and upon disease progression. Depending on resources, storing of DNA from timepoints throughout treatment should also be considered. As molecular MRD monitoring is increasingly incorporated in clinical trials, a practical approach may be to store an aliquot of DNA from each timepoint that MRD is measured. Whilst requiring significant resources to process and store, cryopreserved live tumor cells (stored as peripheral blood/bone marrow aspirate mononuclear cells) are highly valuable to functionally study resistance as well as single cell sequencing. Whilst DNA/RNA/cells form a core sample set, consideration of other sample types may also be relevant such as ctDNA (which may be stored as plasma) or nodal tissue.
- *Analysis type* - The laboratory analyses should be preplanned but also allow for exploratory adjustment in the course of the studies, and need to be adapted to the specific treatment regimens (types of agents and combinations) and study designs (i.e. single vs. multi-arm, dose-finding vs. standard-setting). Both candidate as well as unbiased approaches to resistance mechanisms should be considered. The candidate approach (e.g. targeted BTK/PLCG2 sequencing) is useful to characterize known resistance mechanisms occurring at low level as these approaches typically allow for greater sensitivity. In contrast, unbiased approaches (e.g. whole genome transcriptome sequencing) are valuable for discovery of new resistance mechanisms.

## Summary

Given the high effectiveness and rapid uptake of targeted therapies in CLL, it is important for clinicians to understand the meaning of targeted therapy resistance for their patients. The increasing complexity of this area also highlights the critical role of the diagnostic laboratory in the accurate description, variant curation and effective communication of genomic results issued in the clinical setting (including clinical trials to allow appropriate comparisons and reproducible conclusions). Finally, ongoing research and a deepening of our understanding of resistance mechanisms to targeted therapy gives invaluable insights into disease biology and informs critical elements of clinical trial design and therapeutic drug development.
